# Imaging Based Methods of Liver Fibrosis Assessment in Viral Hepatitis: A Practical Approach

**DOI:** 10.1155/2015/809289

**Published:** 2015-12-08

**Authors:** Hicham Khallafi, Kamran Qureshi

**Affiliations:** ^1^Division of Gastroenterology, Case Western Reserve University, School of Medicine, MetroHealth System, 2500 MetroHealth Drive, Cleveland, OH 44109, USA; ^2^Section of Gastroenterology and Hepatology, Temple University Lewis Katz School of Medicine, Temple University Health System, 3440 N. Broad Street, Philadelphia, PA 19140, USA

## Abstract

Liver fibrosis represents the repair mechanism in liver injury and is a feature of most chronic liver diseases. The degree of liver fibrosis in chronic viral hepatitis infections has major clinical implications and presence of advanced fibrosis or cirrhosis determines prognosis. Treatment initiation for viral hepatitis is indicated in most cases of advanced liver fibrosis and diagnosis of cirrhosis entails hepatology evaluation for specialized clinical care. Liver biopsy is an invasive technique and has been the standard of care of fibrosis assessment for years; however, it has several limitations and procedure related complications. Recently, several methods of noninvasive assessment of liver fibrosis have been developed which require either serologic testing or imaging of liver. Imaging based noninvasive techniques are reviewed here and their clinical use is described. Some of the imaging based tests are becoming widely available, and collectively they are shown to be superior to liver biopsy in important aspects. Clinical utilization of these methods requires understanding of performance and quality related parameters which can affect the results and provide wrong assessment of the extent of liver fibrosis. Familiarity with the strengths and weaknesses of each modality is needed to correctly interpret the results in appropriate clinical context.

## 1. Introduction

Liver fibrosis is a common pathway of liver injury for multiple chronic liver conditions. Chronic viral hepatitis, metabolic, autoimmune, and cholestatic liver diseases amongst others can lead to clinically significant fibrosis. Assessment of the severity of hepatic fibrosis is essential for determining the prognosis of patients with chronic viral hepatitis and estimating the urgency of antiviral therapy [1]. The severity of liver fibrosis and advanced liver disease dictates the need for further evaluations, such as hepatocellular carcinoma surveillance and esophageal varices screening. Although in several cases the diagnosis of advanced liver fibrosis or liver cirrhosis can be made clinically, the liver biopsy has been used as the conventional reference method for staging fibrosis and diagnosis of liver cirrhosis.

Liver biopsy offers histologic visualization of the extent of liver fibrosis and thus is considered the “best” standard for fibrosis staging and is universally accepted; however it is an invasive technique with associated morbidity. Minor complications are relatively frequent and about 25% of patients undergoing liver biopsy have pain in the right upper quadrant or right shoulder after the procedure [2]. Severe complications are uncommon; significant bleeding incidents range from 0.05% to 5.3% and mortality of less than 0.15% was seen in the largest series [3]. The accurate evaluation of fibrosis using liver biopsy is also complicated by sampling error and interobserver variation in staging, particularly when inadequate sampling occurs [4]. A study which included 124 patients with chronic Hepatitis C Virus (HCV) infection who underwent simultaneous laparoscopy guided biopsies of the right and left hepatic lobes showed that 33.1% of subjects were graded to have a difference of at least one stage between the two lobes [5]. Because liver biopsy has several limitations, namely, sampling error and intra- and interobserver variability, the Area Under Receiving Operating Characteristic (AUROC) curve values of >0.9 for liver biopsy could not be attained in clinical studies for fibrosis staging [6], which is a characteristic for an ideal diagnostic test [7]. Rather than the conventional label of being the “gold” standard, liver biopsy is increasingly recognized as the “best” standard for liver fibrosis staging. In addition, performing liver biopsies on a large scale for clinical use may not be a reasonable, cost-effective, or practical approach. Currently, some of the noninvasive methods of liver fibrosis assessment have better diagnostic value than liver biopsy in chronic liver diseases and are safe to perform. As a result, the noninvasive methods are increasingly replacing invasive liver biopsy in clinical practice due to patient wariness of the morbidity associated liver biopsy and physician ease of clinical use with accuracy.

The noninvasive methods of liver fibrosis can be broadly divided into two main groups: serum biomarkers (which require blood sample collection and estimation and calculation of score) and the imaging based methods. In this review, we will focus on imaging based methods and describe the technical aspects, performance accuracy, meaningful utilization, and incorporation in clinical practice of now available elasticity based methods of liver fibrosis assessment (elastography).

## 2. Elastography Imaging

**Figure 1:**
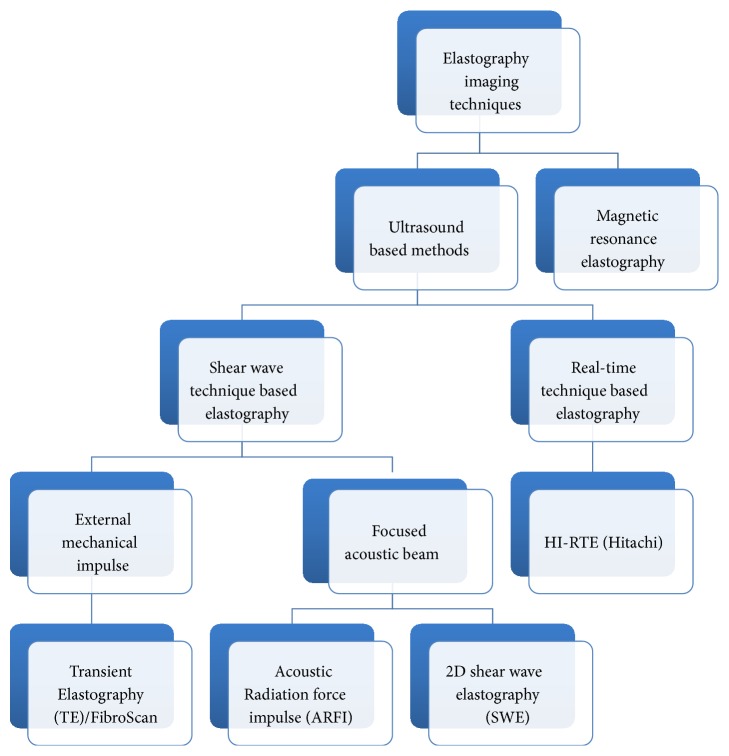
Imaging based elastography techniques for liver fibrosis assessment.

The techniques included can be divided into four main groups: (A) shear wave elastography technique based on mechanically generated impulse that includes Transient Elastography (FibroScan); (B) shear wave elastography techniques based on the acoustic beam that includes 2D shear wave elastography/point shear wave elastography/acoustic radiation force impulse; (C) real-time technique based elastography; and (D) magnetic resonance elastography. These are shown in Figure 1 and overview of comparison of various methods is described in Table 1. It is now recommended that every patient with viral hepatitis should have liver disease staging at least once by noninvasive methods [8]. Transient Elastography (FibroScan) is the most extensively used and validated method for fibrosis staging. Shear wave elastography/acoustic radiation force impulse seems promising; however its incorporation in clinical decision seems to be in early stage with only relatively recent pilot studies. Magnetic resonance elastography is not widely used given the described limitations despite the higher performance. Major advantage of these imaging methods is that these can be conveniently repeated over time in patients who are treated or those who stay on long term treatment for viral hepatitis. The LSM can be followed to monitor for therapy response and improvement of liver fibrosis. In addition, one can expect that the elastography improvement over time will predict improvement in clinical outcomes.

### 2.1. Transient Elastography (TE)

TE or Vibration Controlled Transient Elastography technique is exclusively used in FibroScan which was developed nearly 10 years ago by Echosens [9]. It is now the reference test and is the best validated technique for noninvasive evaluation of liver fibrosis. Its use in the United States was approved by the Food and Drug Administration (FDA) in 2013 for liver fibrosis assessment in HCV patients.

#### 2.1.1. Technical Aspects

TE uses mechanically generated elastic shear wave (SW) and follows its propagation through the liver to measure its speed in a single dimension. The speed of SW in the liver is directly related to its stiffness and the level of fibrosis (elastic modulus) [9]. A probe transducer is used which mechanically generates SW by vibration at the frequency of 50 Hz and is placed on the chest wall in the right ninth to eleventh intercostal space. SW is followed in the cylindrical Region of Interest (ROI) of 1 cm × 4 cm dimension, 2.5 or 6.5 cm below the skin surface to estimate the speed by use of a sensor. A reading is obtained each time button is pressed while probe is in a correct position and is called liver stiffness measurement (LSM), expressed in kilopascals (kPa). The LSMs range from 2.5 to 75 kPa, with mean value around 5 kPa [9]. Accurate result of an examination requires careful interpretation of data from at least 10 valid LSMs. A success rate (the ratio of valid measurements to the total number of measurements) of >60% and an interquartile range (IQR), which reflects variations among measurements, of <30% of the median LSM value (i.e., IQR/median LSM ≤30%) are considered as an adequate study [10]. Table 1 compares certain technical aspects in comparison to other elastography techniques.

#### 2.1.2. Diagnostic Performance and Accuracy

Since the introduction of TE in 2005 in France, its use in early European cohorts of HCV patients has clearly shown that TE can detect clinically significant fibrosis and cirrhosis with a good accuracy and reproducibility [11, 12]. There have been several validation studies in HCV patients indicating that LSM strongly correlates with liver fibrosis on biopsy. Furthermore, in the largest multicenter independent study to date, TE outperformed all other noninvasive tests for the diagnosis of cirrhosis [13]. Similar findings were noted in multiple North American publications. In a recent large United States multicenter cohort study, TE was evaluated in patients with chronic type B and C viral hepatitis (903 patients), and the readings correlated strongly with the fibrosis stage on biopsy with AUROC of 0.89 and 0.92 for significant fibrosis (F ≥ 2) and F4, respectively, with good intra- and interoperator reproducibility (Interclass Correlation (ICC) of 0.95 and 0.99). Liver stiffness cutoff values in this study (kPa) were 8.4 for ≥F2 and 12.8 for F4 [14]. TE generated LSMs have been increasingly applied to other chronic liver diseases besides HCV that include Chronic Hepatitis B Virus (HBV) infection, Nonalcoholic Steatohepatitis (NASH), Alcoholic Liver Disease (ALD), and cholestatic liver diseases [15, 16]. Different liver disease specific stiffness KPa cutoffs were described and are presented in Table 2. Similar to previously noted multiple systemic reviews and meta-analyses, recent large systematic review of published studies between 2001 and June 2011 across chronic liver diseases showed a strong diagnostic accuracy of TE compared to biopsy stages with an AUROC of 0.88, 0.92, and 0.94 for F ≥ 2, F ≥ 3, and F4, respectively [17].

#### 2.1.3. Prognosis and Longitudinal Follow-Up

There is good evidence to support the association between TE measurements and survival. A French study examining 5-year survival in a large cohort of 1457 HCV patients demonstrated that the overall survival using TE for LSM ≤9.5 kPa was 96%; for >9.5 kPa was 77%; for >20 kPa was 66%; for >30 kPa was 57%; for >40 kPa was 47%; and for >50 kPa was 42%. TE had superior diagnostic performance for predicting 5-year survival compared with biopsy [18]. Similar findings were noted in a United States based study with 667 patients with various underlying liver conditions, in which Klibansky et al. reported excellent diagnostic performance of TE for predicting a composite outcome including death, decompensation, and HCC (AUROC of 0.87) [19]. A recent large cohort study (1,025 patients were included) provided strong evidence of the clinical utility of serial TE examinations [20]. Excellent survival from a liver related mortality/transplantation rate of ≤1.2% over 3 years was observed in three groups of patients: (group 1) LSM ≤ 7 kPa regardless of response to antiviral therapy; (group 2) LSM ≥ 7 kPa with sustained virological response; (group 3) LSM 7–14 kPa with a change of LSM ≤ 1 kPa/year. As compared to liver related mortality of 6.6–10.4% in patients with a baseline LSM ≥ 14 kPa or LSM 7–14 kPa with a change of LSM ≥ 1 kPa/year, the worse prognosis (21.4%) was in the group with a baseline LSM ≥ 14 kPa and any increase of LSM over 3 years [20].

#### 2.1.4. Technical Limitations and Training

In the large Castera series (13,369 VCTE examinations reported), the TE examination failure rate and unreliable LSM readings occurred in 3.1% and 15.8%, respectively, and they were mostly associated with BMI greater than 30 kg/m^2^ and operator inexperience [21].

*BMI*. To reduce the number of patients with unreliable readings related to BMI, several probe types have been developed. The XL probe is useful in subjects in whom a valid LSM cannot be obtained with the M probe. Myers et al. reported that the failure rate decreased from 59% for the M probe to 4.9% by using the XL probe for patients with BMI over 40 kg/m^2^ [22]. LSMs taken with the XL probe were noted to be lower than those obtained by the M probe by a median of 1.4 kPa [23]. 

*Operator Experience*. Operator inexperience, defined as <500 examinations, was initially seen to be independently associated with both LSM failure and reading unreliability. Degos et al. demonstrated that there was no difference in the performance of TE between physician performed TE and a trained technician as long as adequate experience was acquired [13]. Kettaneh et al. demonstrated that a reasonable performance for the diagnosis of cirrhosis could be achieved with as few as 50 examinations; the authors conclude the FibroScan may be used in nonspecialized units [24]. United Stated FDA and FibroScan manufacturer agreement requires that an operator undergo a training course and perform a minimum of 10 cases under supervision of a proctor before getting certified to use the device independently. 

*Other Factors*. Liver stiffness is not synonymous with fibrosis and any process that may change hepatic viscoelastic properties can affect LSM readings and lead to overestimation of liver stage. The most important LSM modifying factors include acute inflammation (elevated ALT) [25], extrahepatic cholestasis [26], passive cardiogenic congestion [27], and food intake [28]. Arena et al. showed that after a meal the liver stiffness returned to baseline levels within 120 min in all patients independent of fibrosis stage [28]. Patients without significant fibrosis but high levels of ALT could get LSM within the range for cirrhosis. This was also noted in a large Canadian multicenter study in which the authors reported that, for every 100 IU/mL increase in ALT, LSM increased by 1.1 kPa [29].

#### 2.1.5. Access and Reimbursement in the United States

Since January 2015, a new code 91200 was added for liver elastography performed via mechanically induced SW technique, such as vibration. The code includes coverage for examination and interpretation. Current status shows that only the TE/FibroScan is billable and has been reimbursed. Some insurers may require prior authorization. Medicare reimbursement was reported to approach $134.8, while the additional facility fees were not included.

#### 2.1.6. Clinical Utilization in Viral Hepatitis

TE is well validated in viral hepatitis and its diagnostic performance was found to be equally powerful in HBV, HCV, and HIV-HCV coinfection. However, it is important to use the LSM obtained via TE in appropriate clinical context. The following provides a useful checklist prior to interpretation and use of TE readings. 

*Transient Elastography Clinical Use Checklist for Reliable Interpretation of Results*

- * Operator experience*:
  - Ideally more than 100 examinations.
  - Standardized protocol of examination.
- * Patient characteristics*:
  - Patient diagnosis: HCV, HBV, NASH, or other liver diseases (for use of different cutoffs for diagnosis of cirrhosis).
  - Obesity: use of XL probe (2.5 MHz transducer) for BMI > 30 kg/m^2^ or for thick chest wall (skin to liver surface distance >2.5 cm).
  - Absence of ascites: on clinical or radiologic examination.
  - Fasting status for at least 2 hours.
  - Absence of active alcohol use.
  - Absence of right heart failure/hepatic congestion.
- * Biochemical parameters*:
  - ALT levels: should be less than 5 times ULN for reliable readings, also for use of ALT levels based cutoffs (for HBV).
  - Hepatitis B serology: e antigen status (HBV).
  - Serologic noninvasive fibrosis testing: for intermediate results (HCV).
- * Reliable readings*:
  - Valid readings (shots): at least 10.
  - Success rate (valid shots/total number of shots) of >60%.
  - Variability in valid shots reading (IQR/median LSM) of less than 30%.

It is important to recognize that the clinical confounding variables in clinical decision-making are important (obesity, inflammation, and cholestasis). This entails that a physical examination is done including BMI estimation and assessment of presence of ascites; liver functional and biochemical testing (ALT, alkaline phosphatase) should be available at the time of LSM evaluation. Patient should be fasting for at least 2 hours prior to performing the test. LSMs are a section of the overall diagnosis process and must take into account all the disease clinical, laboratory, and radiological findings. Appreciating the TE intrinsic validities (positive and negative predictive value and AUROCs) and the pretest probability of advanced fibrosis would facilitate the decision-making process. 

**Figure 2:**
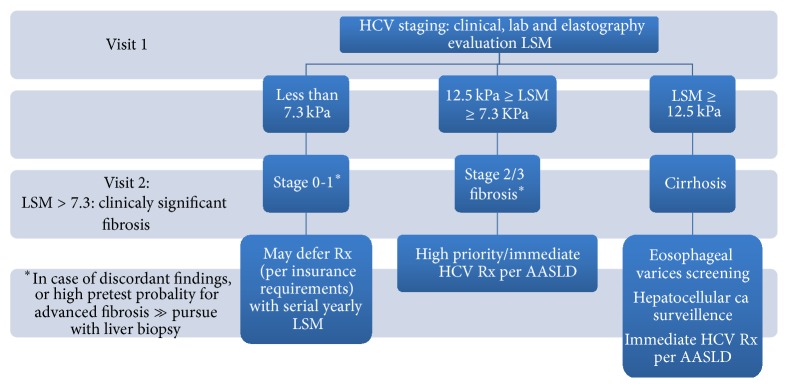
Algorithm for the use of TE for liver staging in HCV and HIV-HCV coinfection.

*HCV including HIV-HCV Coinfection*. TE can identify advanced fibrosis with an excellent accuracy with AUC > 0.9, which was replicated across multiple cohorts, and particularly for the diagnosis of cirrhosis. TE mainly categorizes HCV patients in 2 subsets distinguishing patients with cirrhosis from patients with early fibrosis. Patients in the intermediate fibrosis range may need liver biopsy if accurate staging allocation is needed. Multiple clinical decision algorithms have been developed and can significantly reduce the need for liver biopsy. Boursier et al. showed that biomarkers noninvasive tools in conjunction with FibroScan liver stiffness evaluation could obviate the need for liver biopsy with over 86.7% accuracy [30]. One suggested algorithm for staging of liver disease in HCV is shown in Figure 2. The current recommendations for treatment of HCV differ considerably between healthcare systems and insurance policies. TE can be used to prioritize patients for HCV therapy based on liver disease stage. TE should be combined with serologic noninvasive fibrosis assessment tests to increase the diagnostic accuracy for significant fibrosis and thus initiation of therapy. Once access to care for HCV widens to nonliver or infectious disease providers TE can be used to estimate the presence of cirrhosis and thus referral for specialized care can be suggested [31]. Studies have confirmed histologic improvement and thus clinical outcomes in patients who are cured of HCV infection after therapy [32, 33]. Liver elasticity improves with HCV cure and can be measured as LSM by TE. Several studies showed improvement in LSM after HCV treatment [34, 35]. However, care should be taken while interpreting the follow-up results of TE, as improvement in ALT with HCV treatment will have an impact on LSM. The clinical utility of improvement in LSM after SVR has a little clinical significance in patients without cirrhosis; however the determination of cirrhosis regression after SVR in HCV treated patients could be of important clinical implication. The study that evaluated the cirrhosis estimation showed sensitivity of 61% when the usual cutoff of 12 kPa for cirrhosis was used [36] in patients with cirrhosis who achieved SVR after treatment. This indicates that TE may not be a good tool to estimate cirrhosis regression in HCV patients after SVR, and the time interval between LSM assessments after obtaining SVR is not yet tested. TE showed good predictability, however, in regard to liver related outcomes in HCV patients. In patients who were untreated, a large study showed that gradually rising LSM by 1 kPa per year in HCV patients portends poor clinical outcomes [20]. Other studies were also able to stratify HCV patients based on LSM cutoff values as those with increased risk of long term liver related outcomes [18, 19]. 

*HBV*. Overall TE performs better than the serologic noninvasive methods for diagnosing advanced liver fibrosis in HBV infected patients [37]. Chronic HBV infection has various phases of liver injury, inflammation, and viral replication during a lifetime and TE can be used effectively to estimate the extent of liver fibrosis. TE can yield falsely high readings in patients with elevated liver enzymes (ALT levels), either during active hepatitis phase or during reactivation flares and even 3–6 months after ALT normalization following an acute exacerbation of chronic HBV infection [38]. For HBeAg negative patients who are in immune inactive phase (inactive carriers) TE showed steady results [39] as compared with other methods and LS < 5-6 kPa suggested absence of or minimal fibrosis [40]. LSM readings higher than 12 kPa have good predictability to diagnose cirrhosis in HBV inactive carriers [41], while intermediate LSM values should be followed up with a liver biopsy for correct estimation of liver fibrosis [42]. For HBeAg negative patients in immune active phase and fluctuating or persistent borderline HBV viremia TE may be preferred over performing a liver biopsy to diagnose cirrhosis [43] or advanced fibrosis [42], respectively. Among HBeAg positive patients with normal or mildly elevated ALT, using TE can be helpful in estimation of liver fibrosis in adults to distinguish between immune tolerant phase and advance liver disease secondary to undiagnosed immune active phase of liver injury [44]. The estimation of liver fibrosis by TE can avoid liver biopsy in many patients and guide initiation of antiviral therapy in a timely manner in chronic HBV infection. TE should be considered to diagnose cirrhosis in patient and hence to start therapy with chronic HBV infection and clinical suspicion of advanced liver disease despite normal ALT. Long term antiviral therapy has shown resolution of liver fibrosis/regression of cirrhosis in HBV infected patients [45]. Several studies have shown that TE can be used for follow-up and documentation improvement in liver fibrosis while on HBV therapy [46, 47]. The follow-up LSM readings should be compared with the baseline reading taken once ALT normalize after introduction of antivirals to avoid confounding effect of raised ALT on TE readings [48]. The prognostic utility of LSM obtained by TE in HBV patients in regard to liver related outcomes and survival is being evaluated. One study showed increased risk of HCC in HBV patients who were determined to have cirrhosis by TE assessment [49].

### 2.2. 2D Shear Wave Elastography (SWE)

Cleared by the FDA in December 2014, SWE technique is based on the combination of SW induced by multiple focused acoustic beams and a very rapid acquisition of ultrasound images (up to 20,000 images per second). In contrast to TE, an acoustic SW is generated and evaluated in SWE which can be coupled to the standard liver ultrasound examination for stiffness measurements. Multiple ultrasound systems that have SWE capabilities are commercially available in the United States, including those by Supersonic Imagine, SA (Aix-en-Provence, France; Ultrafast), and Logiq E9 ultrasound system (GE Healthcare, WI, USA).

#### 2.2.1. Technical Aspects

This application is able to capture the propagation of the resulting SW in real time and in a large area of liver parenchyma, developing real-time color-coded elasticity imaging. Quantitative measurements can be performed in the color window by placing one or more ROI inside the sample box. The ROI of SWE is fan-shaped and larger than other modalities (up to 50 mm × 50 mm). Results are given in m/s or kPa [58]. During the B-mode sonographic examination of the liver, a standardized ROI box is positioned in a predetermined anatomical site within the liver parenchyma for evaluation. The SW is generated from a focused ultrasound beam in the vicinity of the designated ROI. The velocity of the wave propagation, expressed in meters per second (m/s) or kilopascals (KPa), is calculated and allows LSM. The measurements are not limited by the presence of ascites as the ultrasound beam generating the SW propagates through fluids. The stiffer the liver is, the higher the recorded SW velocity is [59].

#### 2.2.2. Diagnostic Performance and Accuracy

As the application of SWE to liver fibrosis is a relatively newer approach, there are only a few studies evaluating its accuracy in liver diseases (Table 3). Poynard et al. in the largest liver SWE study to date (422 patients) showed the performance of SWE for the diagnosis of cirrhosis was similar to those of TE except in patients with ascites [52]. The better performance of SWE in patients with ascites may not be important clinically. Another recent single center prospective study with 349 consecutive patients with chronic liver diseases compared the liver stiffness measurements obtained by SWE and TE with liver biopsy [53]. In this study AUROCs of SWE, TE were, respectively, 0.89, 0.86 for the diagnosis of mild fibrosis; 0.88, 0.84 for the diagnosis of significant fibrosis; 0.93, 0.87 for the diagnosis of severe fibrosis; 0.93, 0.90 for the diagnosis of cirrhosis. SWE had a higher accuracy than TE for the diagnosis of severe fibrosis (*⩾*F3) but no statistically significant difference was observed for the diagnosis of mild fibrosis and cirrhosis [53]. The initial United States study was a pilot program that was recently published in September 2014. In this prospective study of 50 patients with chronic liver disease, the SWE measurements were compared to liver biopsy with a good accuracy for advanced fibrosis [60]. SWE is a new approach that has been applied to liver stiffness measurement. However its accuracy and performance in liver diseases need further validation in larger multicenter studies.

#### 2.2.3. Technical Limitations

With SWE only a few diseases specific LSMs are reported. The bulk of the studies have been performed in patients with HCV; therefore these cutoffs may not be applicable to other liver conditions. Only small series of patients with NAFLD have been reported and few studies addressing the confounding factors have been published. Another challenge in interpreting and obtaining data is a lack of standardization.

### 2.3. Acoustic Radiation Force Impulse (ARFI)

Cleared by the FDA in 2013, ARFI is a quantitative technique that provides a single dimensional LSM of predetermined sample box in liver. Similar to SWE, ARFI can be performed at the time of the standard ultrasound evaluation (Table 3). The ROI is smaller than SWE: 1 × 0.5 cm rectangle. The LSM is expressed in m/s, which is reflective of the SW velocity travelling perpendicular to the source [61]. ARFI is developed by Philips Healthcare (Bothell, WA, USA; ElastPQ) and Siemens Medical Solutions (Mountain View, CA, USA; Virtual Touch Tissue Quantification (VTTQ)).

#### 2.3.1. Diagnostic Performance and Accuracy

ARFI was initially validated in a large international multicenter study (10 centers, 5 countries) of 914 HCV patients [55]. The ARFI was compared to liver biopsy and TE; the LSM obtained by the ARFI correlated with Metavir stage with AUROC of 0.792 and 0.842 for significant fibrosis (F ≥ 2) and F4, respectively [55]. LSM cutoff values in this study were 1.33 m/s for ≥F2 and 1.55 m/s for F4. The correlation with histological fibrosis was not significantly different for TE in comparison with ARFI; however TE was better for predicting the presence of liver cirrhosis (*P* = 0.01) than ARFI. A meta-analysis which included 13 studies with 1163 patients and various chronic liver diseases compared ARFI to liver biopsy and TE [56]. The authors showed that ARFI is a good method for assessing liver fibrosis, with AUROC of 0.85 for detecting significant fibrosis and 0.93 for diagnosing cirrhosis with a predictive value similar to TE. Similarly, another meta-analysis, the largest to date (36 studies with 3,951 patients), showed the diagnostic accuracy expressed as AUROC of 0.84 and 0.91 for significant fibrosis and cirrhosis, respectively [57]. Overall, the review of the major published meta-analyses suggests that ARFI is a reliable method for the diagnosis of advanced fibrosis/cirrhosis and likely comparable to TE. However, a wide range of mean values defining cirrhosis was noted contrasting with a narrower range of fibrosis stages cutoffs with FibroScan.

### 2.4. Real-Time Technique Based Elastography (RTE)

This method developed initially by Hitachi Medical Systems, E-Mode, as incorporated into a conventional ultrasound machine is based on strain imaging induced by manual compression. It is a nonquantitative technique which is less accurate in staging liver fibrosis than TE. This technique is not FDA cleared or approved for fibrosis staging.

### 2.5. Magnetic Resonance Elastography (MRE)

MRE was first introduced in the United States by GE Healthcare, based on technology developed at Mayo Clinic (Rochester, MN), and was cleared by the FDA in 2009. This technique is also available on other commercial MRE configurations including Philips Healthcare (2014) and Siemens Medical Solutions (2012).

#### 2.5.1. Technical Aspects

MRE can be coupled to the conventional liver MR imaging protocol and involves 3 steps [62]: (1) propagation of SW within the liver from a driver source generating a continuous 60 Hz acoustic vibration; (2) imaging the propagating SW in the liver using a MRI sequence with motion-encoding gradients; and (3) processing the information in the wave images with an inversion algorithm to generate quantitative elastogram/stiffness maps that measures stiffness in kPa. ROI is placed on the stiffness maps to obtain stiffness values. The ROI permits a larger volume of liver to be sampled. The mean stiffness values reported in the literature range from 2.05 to 2.44 kPa, and the range of normal liver stiffness is determined to be between 1.54 and 2.87 kPa [63].

#### 2.5.2. Diagnostic Performance and Accuracy

In a recent published systematic review and meta-analysis (12 studies with 697 patients with chronic liver diseases), the diagnostic performance of MRE was comparable, if not superior, to that of TE and ARFI [64]. The overall diagnostic accuracy of MRE for discriminating advanced fibrosis (> stage 3) was noted to be excellent with an AUROC of 0.93. The mean AUROC values (and 95% confidence intervals) for the diagnosis of any (≥stage 1), advanced fibrosis (≥stage 3), and cirrhosis were as follows: 0.84 (0.76–0.92), 0.93 (0.90–0.95), and 0.92 (0.90–0.94), respectively [64]. In addition, the authors performed a subgroup analysis; the Body Mass Index (BMI), the degree of liver inflammation, and the underlying etiology of the liver disease did not influence the accuracy of the MRE [64]. Similar results were noted in another meta-analysis in which 11 MRE studies (982 patients) and 15 ARFI studies (2,128 patients) were selected and modalities were compared [65]. Authors concluded that the MRE is more accurate than ARFI particularly in diagnosing early stages of hepatic fibrosis; AUROCs for MRE staging fibrosis were 0.94, 0.97, 0.96, and 0.97 for F1–F4, respectively, whereas AUROCs for ARFI staging were 0.82, 0.85, 0.94, and 0.94 for F1–F4, respectively [65]. Excitingly, MRE consistently has been reported as a highly accurate, noninvasive technique for the diagnosis and staging of liver fibrosis and is likely superior to US based techniques across all published studies. This has been noted across most of the liver diseases.

#### 2.5.3. Technical Limitations

As a new technique, many MRE related studies may not have captured all the potential confounding factors. High hepatic iron content may explain the failure rate noted in early MRE evaluations (signal-to-noise limitations). Patient related factors (inadequate breath hold/claustrophobia) and the cost may be additional limitations to the overall acceptance.

## 3. Conclusion

There is an increasing evidence for the diagnostic and prognostic utility of noninvasive methods of estimation of liver fibrosis and cirrhosis in patients with viral hepatitis. It is estimated that the use of noninvasive imaging based methods to assess liver fibrosis will increase tremendously in the near future. Accurate clinical use and understanding of reported findings will help in patient care and reduce the number of and thus morbidity associated with liver biopsies performed. In addition, imaging based methods have greater diagnostic utility than liver biopsy and can effectively be for follow-up assessments and they might have a role in prognostication of clinical liver related complications and mortality.

## Figures and Tables

**Table 1:** Salient features of technical aspects of liver elastography modalities.

Technique	TE	ARFI	SWE	RTE	MRE
Liver morphologic examination/image	Unable because of lack of visualization	Available as integrated with ultrasound	Available as integrated with ultrasound	Available as integrated with ultrasound	Best as integrated with MRI

Type of force	Dynamic	Dynamic	Dynamic	Static	Dynamic

Applied Force	External mechanical impulse	Internal acoustic beam	Multiple internal acoustic beams	Heart beat	External driver device

Region of Interest (ROI)	Monodimensional ROI: 1 × 4 cm area	Monodimensional ROI: 1 × 0.5 cm	Multiple real-time color-coded elasticity imaging: 2D images ROI: up to 5 cm × 5 cm	Not a quantitative approach: “Relative Elasticity”	Evaluates the entire liver with 2D-3D images

Measurement results (LSM)	Quantitative results displayed in kPa	Quantitative results displayed often in m/s	Quantitative results displayed in kPa or m/s at a wide range of values	Qualitative	Quantitative results displayed in kPa

**Table 2:** Diagnostic use of Transient Elastography in literature.

	Patients (*n*)	Diseases	AUROC		Cutoff kPa	
			≥F2	= F4	≥F2	F4
Ziol et al. [12]	251	HCV	0.79	0.97	8.8	14.6
Castéra et al. [10]	183	HCV	0.83	0.95	7.1	12.5
Marcellin et al. [15]	173	HBV	0.81	0.93	7.2	11.0
Wong et al. [16]	246	NASH	0.80	0.94	7	10.3
Fraquelli et al. [50]	200	All liver diseases	0.84	0.90	7.9	11.9
Degos et al. [13]	1839 Multicenter	All liver diseases	0.76	0.90	5.6	12.9
Afdhal et al. [14]	907 Multicenter	HCV/HBV	0.89	0.92	8.4	12.8
Steadman et al. [17]	Meta-analysis	All liver diseases	0.88	0.94	—	—

**Table 3:** Diagnostic use of acoustic impulse based techniques of elastography in literature.

	Patients (*n*)	Liver disease	AUROC		Cutoff KPa (SWE) and m/s (ARFI)	
			≥F2	= F4	≥F2	= F4
Ferraioli et al. (SWE) [51]	138 Single center	HCV	0.92	0.98	7.1	10.4
Poynard et al. (SWE) [52]	422 Single center	HCV	0.66	0.74	8.8	14.5
Cassinotto et al. (SWE) [53]	349 Single center	All liver diseases	0.88	0.93	8	10.7
Friedrich-Rust et al. (ARFI) [54]	518	All liver diseases	0.87	0.93	1.35	1.8
Sporea et al. (ARFI) [55]	914	HCV	0.79	0.84	1.33	1.55
Bota et al. (ARFI) [56]	1163	All liver diseases	0.85	0.93	1.31	1.80
Nierhoff et al. (ARFI) [57]	3,951	All liver diseases	0.84	0.91	1.37	1.87
